# LB3. Baricitinib plus Standard of Care for Hospitalized Adults with COVID-19 on Invasive Mechanical Ventilation or Extracorporeal Membrane Oxygenation: Results of a Randomized, Placebo-Controlled Trial

**DOI:** 10.1093/ofid/ofab466.1644

**Published:** 2021-12-04

**Authors:** E Wesley Ely, Athimalaipet V Ramanan, Cynthia E Kartman, Stephanie de Bono, Ran Liao, Maria Lucia B Piruzeli, Jason D Goldman, José Francisco Kerr Saraiva, Sujatro Chakladar, Vincent Marconi

**Affiliations:** 1 Eli Lilly, Nashville, Tennessee; 2 University of Bristol, Bristol, England, United Kingdom; 3 Eli Lilly and Company, Indianapolis, Indiana; 4 Swedish Medical Center, Seattle, WA, USA, and Division of Allergy and Infectious Diseases, University of Washington, Seattle, Washington; 5 IPECC - Instituto de Pesquisa Clínica de Campinas, Campinas, Sao Paulo, Brazil; 6 Atlanta VA, Atlanta, GA

## Abstract

**Background:**

Interventions to reduce mortality in critically ill patients with COVID-19 are a crucial unmet medical need. Baricitinib (BARI) is an oral, selective Janus kinase (JAK)1/JAK2 inhibitor with efficacy in hospitalized adults with COVID-19. Treatment with BARI 4-mg was evaluated in critically ill adult patients with COVID-19 with baseline need for invasive mechanical ventilation (IMV) or extracorporeal membrane oxygenation (ECMO).

**Methods:**

COV-BARRIER (NCT04421027) was a randomized double-blind, placebo-controlled trial in patients with confirmed SARS-CoV-2 infection and elevation of ≥ 1 serum inflammatory marker. In this newly completed substudy, enrolled participants (not previously reported) from 4 countries on IMV or ECMO at study entry were randomly assigned 1:1 to once-daily BARI 4-mg or placebo (PBO) for up to 14 days plus standard of care (SOC), which included baseline systemic corticosteroid use in 86% of patients. The prespecified exploratory endpoints included all-cause mortality and number of ventilator-free days (VFDs) through Day 28.

**Results:**

Characteristics for 101 participants are shown in Table 1.

Treatment with BARI significantly reduced all-cause mortality by Day 28 compared to PBO [39.2% vs 58.0%, respectively; hazard ratio (HR) = 0.54 (95%CI 0.31, 0.96), p=0.030, relative risk (RR) = 0.68 (95%CI 0.45, 1.02); Figure 1A]. One additional death was prevented for every six BARI-treated patients. Significant reduction in mortality was also observed by Day 60 [45.1% vs 62.0%; HR = 0.56 (95%CI 0.33, 0.97), p=0.027, RR = 0.73 (95%CI 0.50, 1.06); Figure 1B].

Patients treated with BARI showed a numerical reduction in the duration of IMV and duration of hospitalization vs PBO and more BARI treated patients recovered (Table 2). No new safety findings were observed (Table 2).

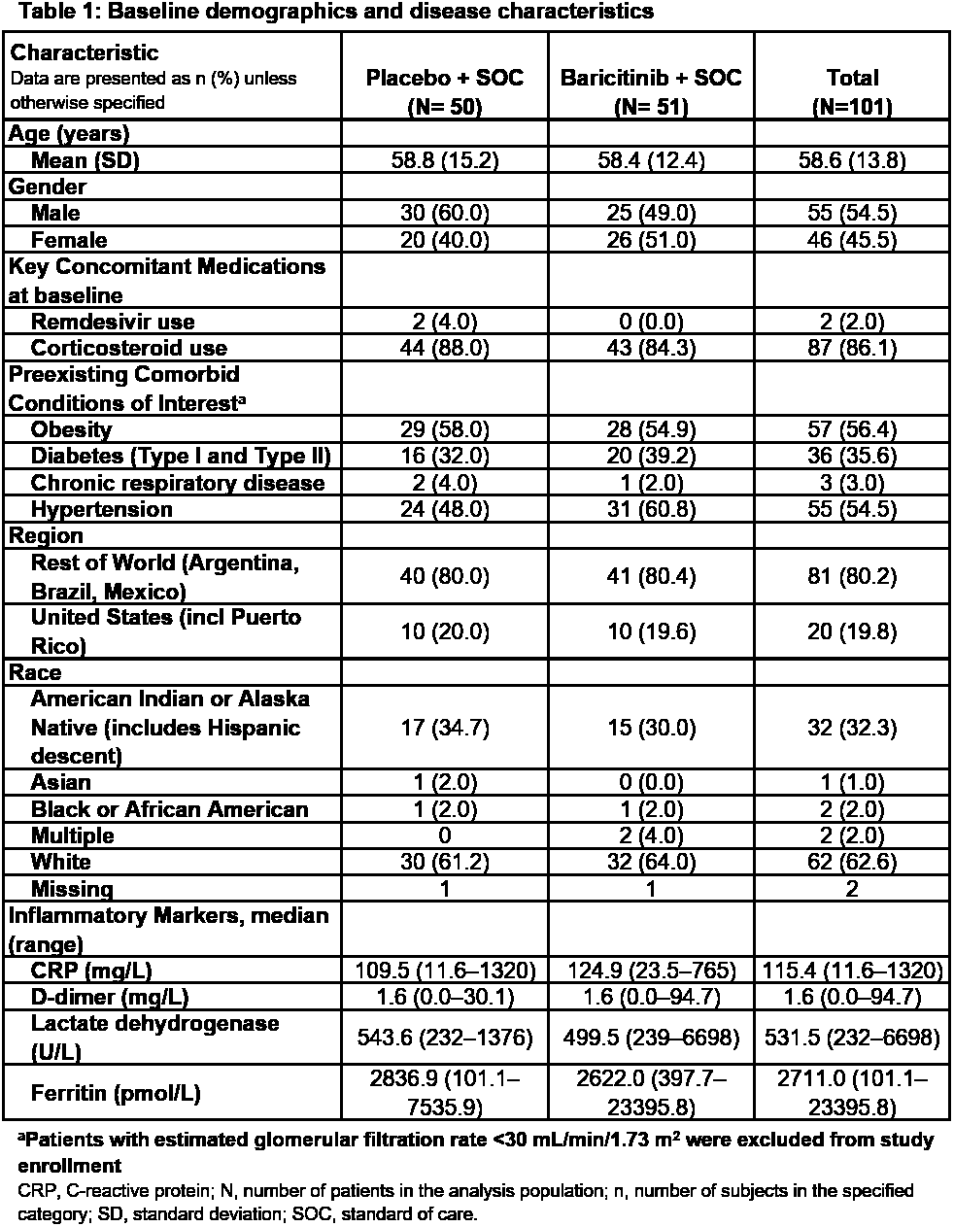

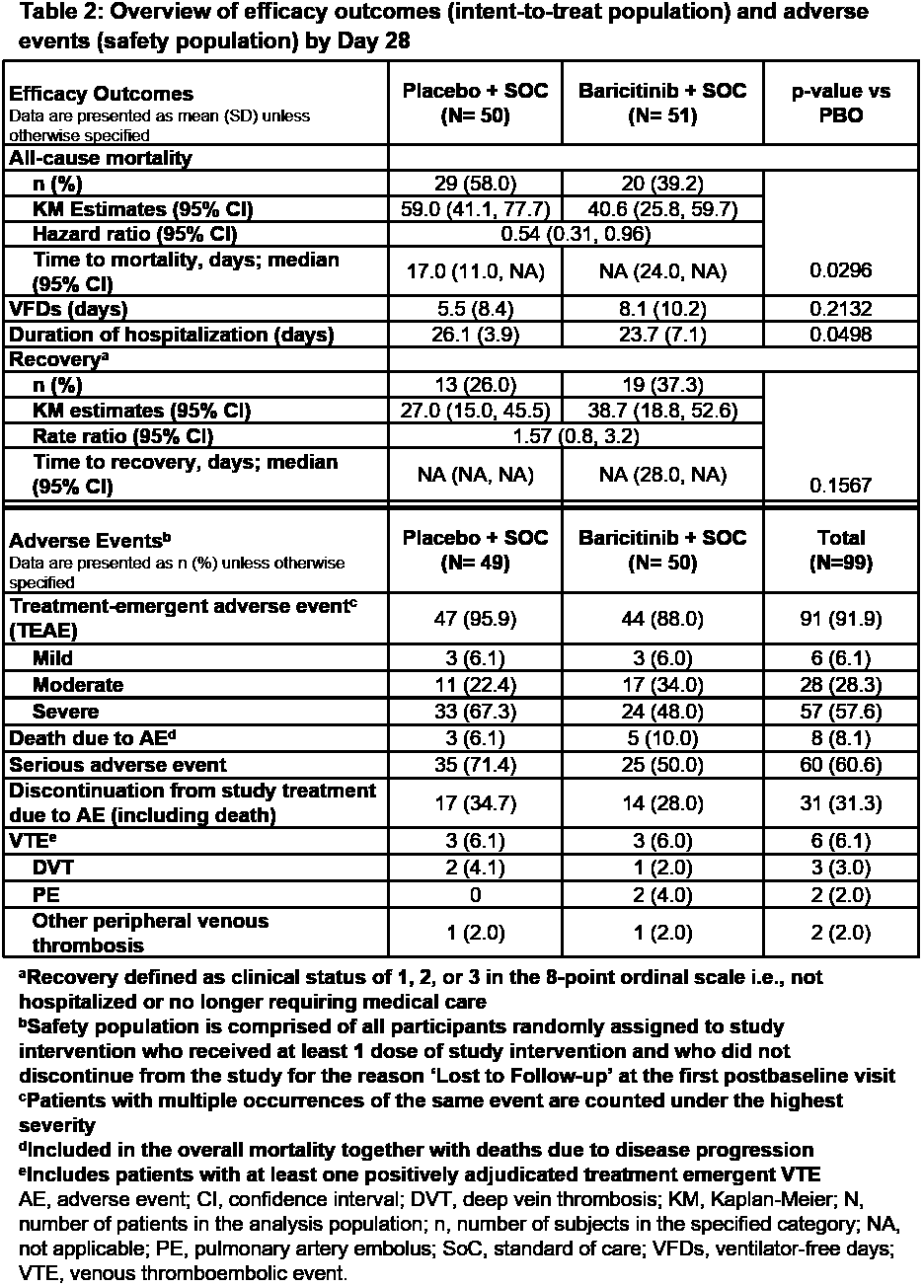

**Conclusion:**

Treatment with BARI+SOC (corticosteroids) resulted in an absolute risk reduction in mortality of 19% at Day 28 and 17% at Day 60 in patients with COVID-19 who were on IMV or ECMO at enrollment. These results are consistent with the reduction in mortality observed in the less severely ill hospitalized patients in the primary COV-BARRIER study population.

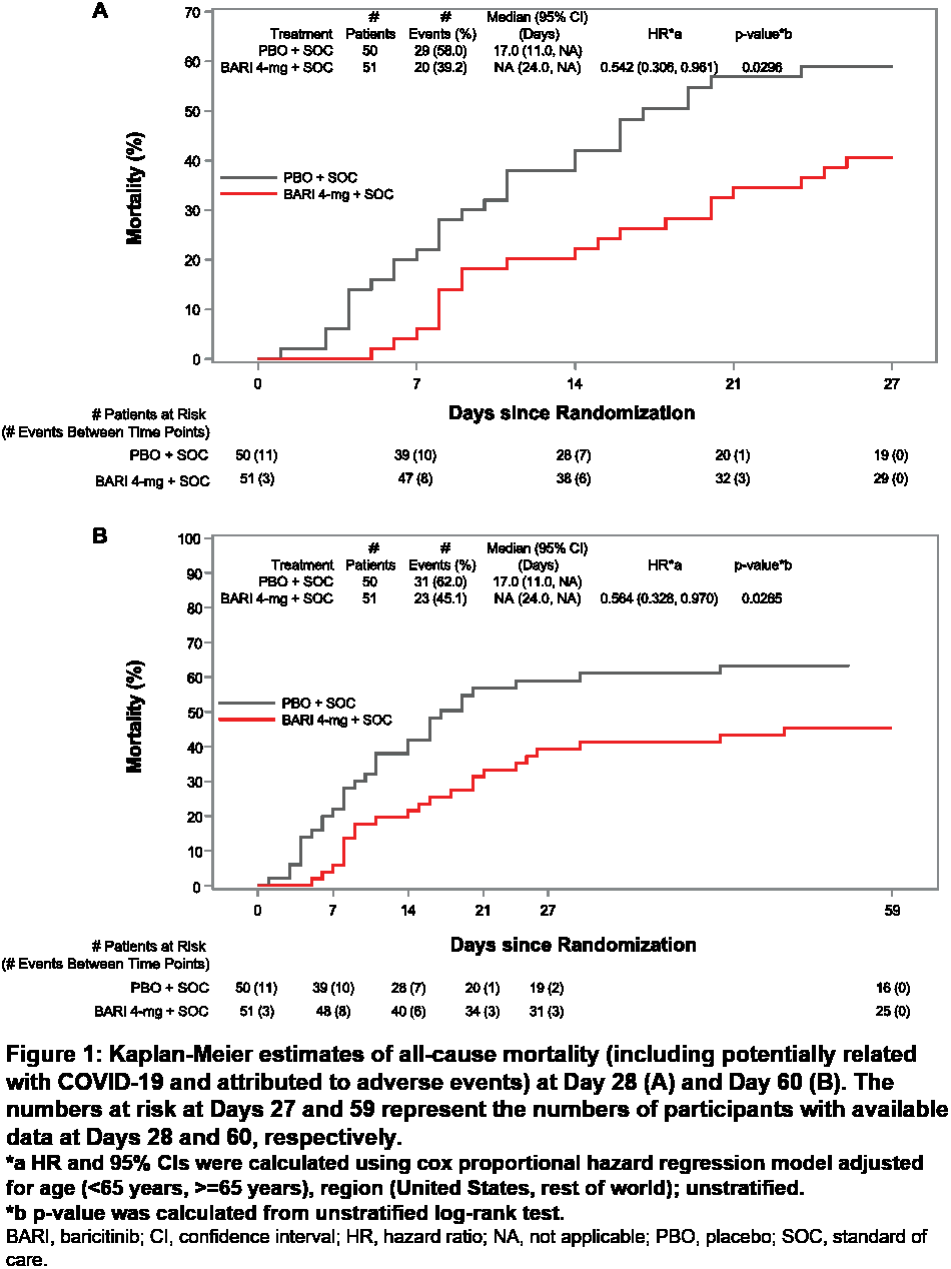

**Disclosures:**

**E. Wesley Ely, MD**, **CDC** (Grant/Research Support)**Eli Lilly** (Other Financial or Material Support, Unpaid consultant)**NIH** (Grant/Research Support)**VA** (Grant/Research Support) **Athimalaipet V. Ramanan, FRCP**, **AbbVie** (Consultant, Speaker’s Bureau)**Eli Lilly and Company** (Consultant, Grant/Research Support, Speaker’s Bureau)**Novartis** (Consultant, Speaker’s Bureau)**Pfizer** (Consultant, Speaker’s Bureau)**Roche** (Consultant, Speaker’s Bureau)**Sobi** (Consultant, Speaker’s Bureau)**UCB** (Consultant, Speaker’s Bureau) **Cynthia E. Kartman, RN BSN**, **Eli Lilly and Company** (Employee, Shareholder) **Stephanie de Bono, MD PhD**, **Eli Lilly and Company** (Employee, Shareholder) **Ran Liao, PhD**, **Eli Lilly and Company** (Employee, Shareholder) **Maria Lucia B Piruzeli, MD**, **Eli Lilly and Company** (Employee, Shareholder) **Sujatro Chakladar, PhD**, **Eli Lilly and Company** (Employee, Shareholder) **Vincent Marconi, MD**, **Bayer** (Consultant, Scientific Research Study Investigator)**Eli Lilly** (Consultant, Scientific Research Study Investigator)**Gilead Sciences** (Consultant, Scientific Research Study Investigator)**ViiV** (Consultant, Scientific Research Study Investigator)

